# Comparison of burr hole drainage and craniotomy for acute liquid epidural hematoma in pediatric patients

**DOI:** 10.1007/s00381-023-06258-8

**Published:** 2023-12-21

**Authors:** Haozhi Ma, Qunjian Cui, Bo Wang, Junfeng Chen, Zhixuan Wei

**Affiliations:** 1 Xinxiang Medical University, Xinxiang, 453003 China; 2 Neurosurgery of the First Affiliated Hospital of Nanyang Medical College, 46 Chezhan South Road (Intersection of Zhongzhou Road and Chezhan Road), Nanyang City, Henan Province, 473007 China

**Keywords:** Liquid extradural hematoma, Burr hole drainage, Craniotomy, Epidural hematoma, Pediatric EDH

## Abstract

**Purpose:**

To compare the impact of burr hole drainage and craniotomy for acute liquid epidural hematoma (LEDH) in pediatric patients.

**Methods:**

This retrospective study enrolled pediatric patients with LEDH who underwent surgery in the Affiliated Hospital of Nanyang Medical College, China, between October 2011 and December 2019. According to the surgical procedure, patients were divided into the craniotomy group and the burr hole drainage group.

**Results:**

A total of 21 pediatric patients were enrolled (14 males, aged 7.19 ± 2.77 years), including 13 cases in the burr hole drainage group and 8 patients in the craniotomy group. The operation time and hospitalization period in the burr hole drainage group were 33.38 ± 6.99 min and 9.85 ± 1.07 days, respectively, which were significantly shorter than that in the craniotomy group (74.25 ± 9.68 min and 13.38 ± 1.71 days, respectively; all *p* < 0.05). The Glasgow Coma Scale (GCS) score after burr hole drainage was significantly improved than before (median: 15 vs 13, *p* < 0.05). No serious complications were observed in either group; one patient in the craniotomy group developed an infection at the incision point. All patients were conscious (GCS score was 15) at discharge.

**Conclusion:**

Compared with craniotomy, burr hole drainage was associated with better clinical outcomes and early recovery in patients with LEDH.

## Introduction

Head injury is a leading cause of mortality and morbidity in the pediatric population [1]. Children who suffer traumatic brain injuries typically have a worse prognosis than adults. Also, recent studies suggest that children and adolescents have worse post-TBI outcomes and take longer to recover than adults. Epidural hematoma (EDH) is a common type of craniocerebral injury that accounts for 2–3% of all head injuries in infants and children [2]. EDH is caused by the accumulation of blood between the skull’s inner table and the meninges’ stripped-off dural layer.

Rapid diagnosis and evacuation are important for a good EDH outcome [3]. Compared to adults, diagnosis of EDH in children requires specialized knowledge of anatomical location. Computed tomography (CT) scan remains the standard diagnostic tool [4]. In acute phase, hematoma tends to coagulate quickly, and CT manifests high-density signals for such lesions.

Acute liquid epidural hematoma (LEDH) is a special kind of EDH that rarely occurs in the pediatric population [5]. It develops slower than a typical acute EDH, with intracranial hypertension, neurological signs, and symptoms appearing 2 to 3 days after injury. In addition, the bleeding source for LEDH is mostly the plate barrier vessels at the fracture site and possibly the dural venous sinus. The CT images of the pediatric patients included in this study were obtained within 7 h after the injury, during the acute phase. The CT scans of epidural hematoma during the acute phase usually exhibit a high signal intensity; however, in our LEDH patients, predominantly uniform same-density or low-density lesions were observed compared to brain parenchymal and subsequently verified through surgical intervention as non-coagulated fluid. The application of trepanation and drainage remains controversial for LEDH patients requiring surgical treatment [6]. In addition, it is still debatable whether burr hole drainage or craniotomy could be the standard practice to ensure good clinical and early recovery in patients with LEDH.

As the safety and effectiveness of trepanation and drainage for LEDH need further discussion, this study aimed to compare the impact of burr hole drainage and craniotomy for acute LEDH in pediatric patients.

## Materials and methods

### Study design and subjects

This retrospective study included patients diagnosed with LEDH in the neurosurgery department of the Affiliated Hospital of Nanyang Medical College between October 2011 and December 2019. The study was approved by the ethics committee of the Affiliated Hospital of Nanyang Medical College, and written informed consent was acquired from all subjects.

The inclusion criteria were as follows: (1) patients aged ≤ 16 years with high equidensity (> 75%) lesions on plain CT scan; (2) Glasgow Coma Scale (GCS) score ≥ 8, without unstable consciousness or signs of brain herniation such as dilated pupils; and (3) patients with supra-tentorial hematoma ≥ 20 mL, the infra-tentorial hematoma ≥ 10 mL, and the thickness of the hematoma ≥ 1 cm according to the Tada formula. The exclusion criteria were as follows: (1) patients with a history of trauma; (2) patients with complicated dysfunction or injury of important organs such as heart, lung, liver, and kidney; and (3) patients with obvious brain contusion, brain herniation before operation, or coagulation disorders.

### Procedure

#### Craniotomy

The location of the hematoma was identified using cranial CT and a horseshoe incision in the scalp after successful general anesthesia. The skull was then occluded, and the middle temporal or superior temporal gyrus was selected as the entrance point for the hematoma site. External ventricular drainage was performed after the removal of the hematoma. Following removal of the hematoma, a catheter was left in situ, the bone flap was appropriately repositioned, and the surgical incision was sutured. Postoperative antibiotics were administered prophylactically in order to minimize the risk of infection.

#### Burr hole drainage

After successful administration of local anesthesia, the hematoma site was identified through a cranial CT scan. A suitable target at the hematoma’s center was selected to avoid damaging the brain’s major functional areas, blood vessels, and meninges. Using a YL-1 disposable hematoma-crushing puncture needle, the surgeon accessed the hematoma site by drilling through the scalp and skull. A hematoma was aspirated slowly while continuously modifying the direction of the needle until no fluid was aspirated. Then, the hematoma lesion was washed with normal saline. After a 4-h retention period, if no signs of active bleeding from the lesion were observed, urokinase was administered to initiate open drainage. Postoperative imaging was conducted on the fifth day to validate the total elimination of the hematoma, and drainage tubes were extracted once elimination was confirmed.

### Data collection

Demographic and clinical data including gender, age, typical clinical features, white blood cells, platelets, hemoglobin, activated partial thromboplastin time (APTT), prothrombin time (PT), thrombin time (TT), fibrinogen (Fbg), location and volume of hematoma, pre-operative GCS, 72-h post-operation GCS, GCS at discharge, anesthesia time, operation time, hospital stay, and complications were obtained from the electronic medical record system for all patients.

### Statistical analysis

The data was analyzed with SPSS-19.0 (IBM Corp., Armonk, NY, USA). Variables with normal distribution and homogeneity of variance were expressed as mean ± standard deviation (SD) and compared with a *t* test, while variables of skew distribution were represented by median (Q_25,_ Q_75_) and analyzed using the non-parameter rank sum test. A *p*-value < 0.05 was considered to be statistically significant.

## Results

This study included 21 pediatric patients, including 14 males and 7 females, with an average age of 7.19 ± 2.77 years. Among them, 13 patients were treated with burr hole drainage and 8 patients underwent craniotomy. A mild coma (GCS score, 12 points) was reported in 9 cases, hypersomnia (13 points) in 8 cases, lethargy (14 points) in 3 cases, and consciousness (15 points) in 1 case only. The median platelet count was 218 (167, 270) × 10^9^/L, and the median hemoglobin was 128.00 (121.00, 132.00) g/L. Coagulation function indicators, including the median APTT, PT, TT, and Fbg, were 31.72 (28.62, 34.48) s, 11.62 (9.82, 12.97) s, 17.32 (16.23, 19.12) s, and 2.19 (2.01, 2.29) g/L, respectively. CT manifestations of LEDH lesions in the acute phase were isodense to low dense or mostly isodense with a small amount of high density. All 21 patients had a skull fracture and scalp hematoma. Nine LEDHs were located in the frontal region, 2 in the parietal region, and 4 in posterior cranial fossa, and in 3 cases, both temporal and occipital regions were involved (Table 1).

**Table 1:** General information of patients

	***n* = 21**
Age, mean ± SD	7.19 ± 2.77
Gender, *n* (%)	
Male	14 (66.67%)
Female	7 (33.33%)
Typical clinical manifestations, *n*	
Lucid	1
Hypersomnia	14
Lethargy	6
Headache	21
Nausea and vomiting	19
Local scalp swelling	21
Leukocyte, ^10^12^/L, median (Q25; Q75)	10.32 (9.52, 12.83)
Platelet,^10^9^/L, median (Q25; Q75)	218 (167, 270)
Hemoglobin, median (Q25; Q75)	128.00 (121.00, 132.00)
APTT, s, median (Q25; Q75)	31.72 (28.62, 34.48)
PT, s, median (Q25; Q75)	11.62 (9.82, 12.97)
TT, s, median (Q25; Q75)	17.32 (16.23, 19.12)
Fbg, g/L, median (Q25; Q75)	2.19 (2.01, 2.29)
Hematoma site, *n* (%)	
Frontal region	9 (42.86%)
Parietal region	2 (9.51%)
Temporal region	3 (14.29%)
Occipital region	3 (14.29%)
Posterior cranial fossa	4 (19.05%)
Preoperative GCS score, median (Q25; Q75)	13 (13, 14)
Borehole drainage, *n* (%)	13 (61.9%)
Craniotomy, *n* (%)	8 (38.1%)

The operation time and hospital stay in the burr hole group were 33.38 ± 6.99 min and 9.85 ± 1.07 days, respectively, which were significantly shorter than that in the craniotomy group (74.25 ± 9.68 min and 13.38 ± 1.71 days, respectively, all *p* < 0.05, Table 2). The GCS score of all patients at 72-h post-procedure was significantly improved compared to the pre-operative GCS score (*p* < 0.05, Table 2). One patient in the craniotomy group developed a wound infection. However, no acute arterial bleeding or other complications were found in either group. The median GOS calculated at the time of discharge was 15 in both groups, suggesting a good treatment outcome. A statistically significant improvement in GCS of both groups was observed when compared pre-operatively and 72 h postoperatively (*p* = 0.001 and *p* = 0.011, Table 3).

**Table 2:** Comparison of operation, anesthesia, and hospitalization time of different operation methods

	**Burr hole drainage (*n* = 13)**	**Craniotomy (*n* = 8)**	***t*-value**	***p*-value**
Operation duration (min)	33.38 ± 6.99	74.25 ± 9.68	12.21	0.006
Duration of anesthesia (min)	36.54 ± 6.89	79.75 ± 9.33	11.26	0.007
Hospitalization days (d)	9.85 ± 1.07	13.38 ± 1.71	6.52	0.006

**Table 3:** A comparison of preoperative and postoperative GCS scores

	**Preoperative GCS score**	**GCS score 72 h after operation**	***Z*-value**	***p*-value**
Burr hole drainage	13 (12.00;13.00)	15 (14.50;15.00)	− 3.23	0.001
Craniotomy	12 (12.00;13.00)	15 (14.00;15.00)	− 2.55	0.011

### Typical case

**Fig. 1:**
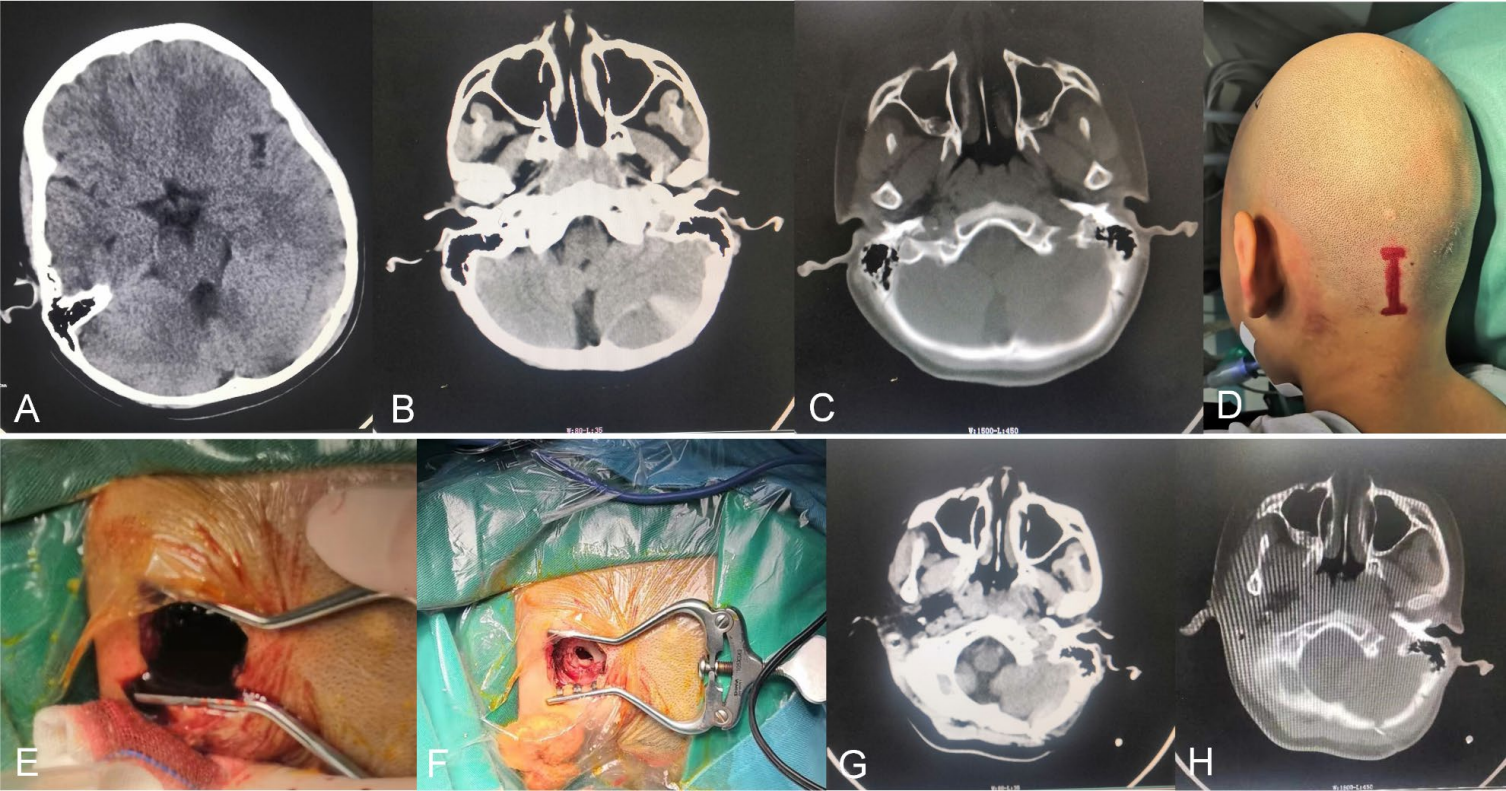
A 9-year-old patient with LEDH. **A** Occipital skin swelling and a small amount of epidural hematoma were found 3 h after injury. **B** The size of the hematoma was increased in the cerebellar hemisphere 7 h after injury. **C** The fracture line of the occipital bone was observed through the bone window. **D** Intraoperative incision. **E** Dark-red hematoma flows out after drilling. **F** After the hematoma had flown out, no CSF came out of the bone hole. **G, H** Postoperative CT scan reexamination showing hematoma elimination

A 9-year-old boy was admitted with the complaint of headache, dizziness, and nausea lasting 3 h after fall. Upon admission, the patient was lethargic, with a GCS score of 13/15 points. The initial CT brain scan showed swelling of the occipital scalp, a small amount of epidural hematoma in the occipital region of brain, and a skull fracture (Fig. 1A). Later, the patients symptoms worsened. The results of repeated CT scans and MRI reexamination of the head at 7-h post-fall showed that the volume of hematoma increased along with a larger portion of isodense hematomas and a small portion of high-density hematomas (Fig. 1B and C). After obtaining informed consent from his guardians, burr hole drainage of hematoma was performed in the emergency operating room. With an appropriate surgical incision (Fig. 1D), a single burr hole was made, and dark red non-clotted hematoma was evacuated (Fig. 1E). No cerebrospinal fluid (CSF) leak was observed (Fig. 1F). The surgery was performed uneventfully, and the patient recovered well. Postoperative CT and MRI examination showed complete hematoma evacuation (Fig. 1G). The postoperative GCS score was 15, and the patient was discharged after 11 days of hospital stay. The patient had no complications 2 months later, and the follow-up examination results were normal.

## Discussion

In the present study, 13 LEDH patients (61.9%) who underwent burr hole drainage showed good postoperative outcomes. No coagulation abnormalities or CSF leaks were recorded in the study participants. Additionally, shorter operative and anesthesia times were recorded compared to standard craniotomy. This study adds significantly to the literature, helps define the curative effect of trepanation and drainage of LEDH, and sets guidelines for its management in children.

LEDH is a rare subtype of EDH found in children. This study aimed to find out the impact of burr hole drainage on LEDH compared to traditional craniotomy in child populations. According to previous research, most children with stable normal conditions, a small hematoma, and no obvious midline structure deviation have a good prognosis [7, 8]. However, for patients with surgical indications, craniotomy is currently the standard surgical treatment [9]. In this study, burr hole drainage performed in 13 pediatric patients for LEDH showed favorable postoperative outcomes. Comparable results have been reported by Habibi et al*.* [10], where 8 cases of LEDH were treated with drilling and drainage, which showed good clinical results. The findings of their study indicated that drilling and drainage might be a more minimally invasive treatment for slowly developing LEDH. Similarly, Ren et al*.* [11] performed trepanation and drainage for 5 cases of postoperative EDH with equal density and achieved good results, finding that the equal density on CT reflected their liquid properties, which favored easy evacuation. Good clinical outcomes have been shown in various studies where burr hole drainage with or without the use of urokinase was employed for traumatic extradural bleeds [12, 13].

Studies have shown that most of the liquefied EDH is associated with coagulation abnormalities, which is not consistent with our findings, where all cases had normal coagulation profiles. Mahajan et al*.* [10] reported two cases of LEDH, indicating that low hemoglobin concentration and low hematocrit were important factors leading to this phenomenon. In our study, the median hemoglobin level of all participants was normal, unlike what Mahajan and his team reported. They also revealed that the mixture of blood and CSF inhibited the coagulation of hematoma and led to fast dissolution. Similarly, other researchers showed that damage to the dura mater and arachnoid membrane after injury could lead to the outflow of CSF and the dilution of hematoma [14]. However, the results of our intraoperative exploration showed no evidence of a dural tear or CSF leak. Ren et al*.* showed that the drilling evacuation could shorten the operation and hospitalization time compared to the craniotomy, resulting in less trauma and a good prognosis [11]. These results are in accordance with our study observations, where significantly reduced anesthesia and operation time were achieved with burr hole drainage compared to the standard craniotomy. Subsequently early recovery and statistically shorter hospital stay were observed.

A few studies have pointed out that most of these hematomas are located near the venous sinus, which may be related to the damage of the venous sinus, leading to serum exudation and contributing to the non-coagulation of hematoma [15]. In our study, only part of the posterior cranial fossa hematoma was located close to the venous sinus, while the rest were located supratentorially away from the venous sinuses. If a patient develops signs of re-bleeding after trepanation and drainage, the standard craniotomy hematoma removal should be used to better control bleeding [16]. As demonstrated in the present study, burr hole drainage without the need for traditional craniotomy would suffice for the management of acute simple LEDH in the pediatric population. This technique has also been performed as a life and time-saving procedure in mass causality scenarios.

The present study has some limitations. First, it was conducted at a single center and on a small sample size (*n* = 21). Consequently, to increase the generalizability of these results, future studies at multiple tertiary centers and with a larger sample are needed to gather sufficient data. Furthermore, the study did not scrutinize the mechanism of hematoma development, which should be further investigated.

## Conclusions

It is paramount to note that rapid diagnosis and timely management of LEDH may reduce mortality and morbidity. Burr hole drainage of hematoma might be an accessible management method for the pediatric population. The specific reasons for developing such hematoma are primarily based on the clinical characteristics of inference; thus, more clinical research support is needed to further elaborate the specific mechanism.

## Data Availability

All data generated or analyzed during this study are included in this published article.
